# An Explainable AI Application (AF’fective) to Support Monitoring of Patients With Atrial Fibrillation After Catheter Ablation: Qualitative Focus Group, Design Session, and Interview Study

**DOI:** 10.2196/65923

**Published:** 2025-02-13

**Authors:** Wan Jou She, Panote Siriaraya, Hibiki Iwakoshi, Noriaki Kuwahara, Keitaro Senoo

**Affiliations:** 1 Faculty of Information and Human Sciences Kyoto Institute of Technology Kyoto Japan; 2 Department of Cardiovascular Medicine Graduate School of Medical Science Kyoto Prefectural University of Medicine Kyoto Japan; 3 Department of Cardiac Arrhythmia Research and Innovation Graduate School of Medical Science Kyoto Prefectural University of Medicine Kyoto Japan; 4 Department of Advanced Fibro-Science Kyoto Institute of Technology Kyoto Japan

**Keywords:** atrial fibrillation, explainable artificial intelligence, explainable AI, user-centered design, prevention, postablation monitoring

## Abstract

**Background:**

The opaque nature of artificial intelligence (AI) algorithms has led to distrust in medical contexts, particularly in the treatment and monitoring of atrial fibrillation. Although previous studies in explainable AI have demonstrated potential to address this issue, they often focus solely on electrocardiography graphs and lack real-world field insights.

**Objective:**

We addressed this gap by incorporating standardized clinical interpretation of electrocardiography graphs into the system and collaborating with cardiologists to co-design and evaluate this approach using real-world patient cases and data.

**Methods:**

We conducted a 3-stage iterative design process with 23 cardiologists to co-design, evaluate, and pilot an explainable AI application. In the first stage, we identified 4 physician personas and 7 explainability strategies, which were reviewed in the second stage. A total of 4 strategies were deemed highly effective and feasible for pilot deployment. On the basis of these strategies, we developed a progressive web application and tested it with cardiologists in the third stage.

**Results:**

The final progressive web application prototype received above-average user experience evaluations and effectively motivated physicians to adopt it owing to its ease of use, reliable information, and explainable functionality. In addition, we gathered in-depth field insights from cardiologists who used the system in clinical contexts.

**Conclusions:**

Our study identified effective explainability strategies, emphasized the importance of curating actionable features and setting accurate expectations, and suggested that many of these insights could apply to other disease care contexts, paving the way for future real-world clinical evaluations.

## Introduction

### Background

While the role of artificial intelligence (AI) in health care is growing, concerns about its reliability and transparency remain largely unaddressed [1]. The opaque nature of AI algorithms challenges trust, particularly in cardiology, where the lack of interpretability in deep learning models limits their adoption and utility [2,3]. This has led to increased interest in developing explainable AI models that offer accurate predictions along with clear explanations of the rationale behind the predictive results [4].

Our study explored the advantages and challenges of using AI models in cardiovascular treatment, focusing on designing an explainable AI system to aid posttreatment care for patients with atrial fibrillation (AF) after catheter ablation. There were >3 million new cases of AF, a common arrhythmic disorder [5], in 2017, with numbers expected to rise due to aging [6]. Although often undetected, AF is a significant risk factor for severe conditions such as stroke or heart failure [7] and can greatly impact patients’ quality of life [5,8]. While catheter ablation is effective, AF can recur after ablation, requiring careful monitoring of risk factors [9].

Several machine learning models, from screening algorithms [10,11] to those predicting AF recurrence after ablation [12], have been developed, but integrating them into health care remains challenging. Physicians often require detailed justifications due to prediction uncertainties. While explainable AI algorithms [5,13] could assist, a more holistic approach is needed in posttreatment scenarios to effectively integrate additional information, which remains poorly understood in real-world care contexts (discussed in the next section).

To better integrate a machine learning model into the ongoing decision-making ecosystem and identify unmet clinical needs beyond performance optimization, we used a user-centered, iterative design approach to develop AF’fective, an explainable AI system for posttreatment monitoring of patients with AF. By conducting semistructured interviews, design sessions, and focus groups with 23 cardiologists, we aimed to identify key principles for the effective design and use of explainable AI in real-life cardiac treatment and management. In this section, we first review the development of explainable AI in relevant fields in the second subsection and summarize our first 2 study stages in the third subsection. We then detail and discuss our final-stage findings and conclude with reflections on the study’s implications and the generalizability of our insights.

### High Accuracy but (Still) Rejected: Machine Learning Models in Cardiology

Cardiology is one field of medicine that has seen extensive use of AI to aid in medical practice. Various machine learning models have been developed and used to assist in a wide range of tasks, from helping predict the prognosis or readmission after heart failure [14,15] to detecting various cardiovascular diseases from medical images [16]. In the area of arrhythmia, previous studies have focused particularly on two key areas: (1) prevention, which involves assisting in the early detection of various arrhythmic conditions; and (2) monitoring, which involves supporting the management of such conditions after treatment [17]. In the preventive area, researchers have shown how machine learning models could be developed and used to detect irregular conditions using electrocardiography (ECG) signals [18] or even through photoplethysmography sensors from commercially available smartwatches [19,20]. In most cases, such models were able to diagnose these irregular conditions with a high degree of accuracy (97%) [20]. However, the potential for a high rate of false positives when implemented through commercial smartwatches has triggered concerns among researchers, and as a result, they have cautioned against using such systems for population-wide screening and emphasized maintaining the 12-lead ECG as the gold standard [21]. Given the prevalence of AF, several of the developed models in this domain have chosen to focus specifically on this condition, with various deep learning ECG models being developed to help screen people for AF, stratify patients based on their risk level, and even predict the chances of the condition occurring in the future (see the study by Sehrawat et al [21] for a comprehensive review).

In the monitoring area, to support posttreatment care, previous studies have developed models capable of predicting patient mortality or echocardiographic response after procedures such as cardiac resynchronization therapy [22]. Other studies have even shown how the risk of recurrence for conditions such as AF could be predicted using patient demographics and 3D computed tomography images of the left atrium [23]. Similarly, several studies have demonstrated how the recurrence of AF or 30-day hospital readmission after catheter ablation can be predicted using various deep learning and non–deep learning strategies (eg, convolutional neural network [CNN] and Extreme Gradient Boosting) [12,13,24]. In other cases, deep learning algorithms have also been developed to detect an AF episode 4.5 minutes before onset, thus enabling prompt interventions to prevent their occurrence [25].

Overall, while the findings of these studies all appear to demonstrate significant potential for implementing predictive models to support AF treatment, we have encountered substantial pushback from clinical partners when attempting to implement such a model in clinical practice. Despite the promising performance of the model in the study by Nishimura et al [26] (area under the curve of 0.72 with 83% sensitivity and 58% specificity), there was still strong resistance from physicians regarding trusting the predicted outcomes. Such hesitation and resistance to adopting machine learning models among clinical staff have also been frequently identified in previous studies [1,27]. Given the delicate and often critical nature of treatment in this field, it is not surprising that the lack of interpretability undermines trust in these models, preventing clinicians from using them in medical treatment (see the study by Petch et al [2]). Moreover, the opaque process raises concerns regarding how mistakes could be rectified, not to mention that it may cause potentially disruptive emotions when discussing such results with patients. Therefore, at present, the high accuracy, accessibility, and (possibly) low cost of machine learning models often do not lead to their adoption in medical practice.

### Explainable AI in AF

Recently, explainable AI has gained attention as a potential approach to address these issues of transparency and interpretability [28-30]. In the context of AF treatment, ECG data are generally used as a main data source for screening irregular rhythm and predicting the risk of AF recurrence, and as such, explainable AI techniques such as Shapley Additive Explanations (SHAP) have been used with the ECG data to identify features contributing to the predictive outcomes [31]. In previous studies, researchers have proposed an explainable deep learning model that not only detects AF but also describes the reasons behind their decisions and visualizes key regions within the ECG signal identified as important predictors using techniques such as Gradient-Weighted Class Activation Mapping (Grad-CAM) or a deep visual attention [29,32,33]. Building upon earlier models, researchers such as Raza et al [34] incorporated federated learning techniques alongside explainable AI to preserve patient ECG data privacy. In more recent studies, explainable models have even been created that are capable of identifying patients at high risk after catheter ablation and highlighting the features (eg, type of AF, age, and left atrial diameter) that the model used to decide on the risk level for each patient [30]. The use of “explainable” models in this manner has been argued to have the added benefit of allowing clinicians to better understand the relationship between each contributing factor and the predicted outcome of an individual case, helping clinicians justify their decisions and treatments more effectively [35].

While explainable AI techniques are believed to enhance the interpretability of algorithms, incorporating them to truly improve patient outcomes in real-life medical contexts remains challenging [36]. Understanding of the strengths and limitations of these approaches in different use cases and how human and AI-based diagnoses can complement each other is still lacking [36]. Deploying these models effectively requires a thorough examination of the patient journey and adapting the explainable strategy accordingly. Recent studies [37,38] have begun to address these issues by examining the use of explainable AI in more realistic settings with various stakeholders to ensure safe adoption in clinical practice.

Overall, although the integration of explainable AI into medical contexts holds great promise, it requires a concerted effort to address technical, ethical, and practical challenges. Insights from near-live and real-life case studies can help develop best practices and trustworthy methods for implementing such systems in clinical settings, thereby enabling users and professionals to fully benefit from AI technology.

### Early Study Stages and Findings in a Nutshell

In this section, we summarize and report the in-depth insights from the 2 early stages of interviews (partially reported as a poster in the study by She et al [39]) and group design sessions to conceptualize and pilot-test our design strategies and prototype. We chose to include some key findings from these early stages to enhance readers’ understanding of our design decisions in developing the final prototype for evaluation.

#### Stage 1

##### Overview

In the first stage, we conducted semistructured interviews with physicians to gain insights into their experiences and best practices for posttreatment care of patients with AF. Physicians were asked to share their positive and negative experiences, communication strategies, and effective procedures for monitoring posttreatment patients with AF. To sensitize and prepare participants, we provided a 1-week workbook with reflection exercises sent 1 to 2 weeks before the interview. Each interview lasted approximately 45 minutes and was fully recorded with the consent of the physicians.

##### Participants

We invited 8 physicians from different specialties, such as emergency medicine, internal medicine, and cardiology, to join us for interviews at Kyoto Prefectural University of Medicine in Japan. We made sure to include a mix of experience levels and backgrounds in cardiology to obtain insights that could be more broadly generalized to other medical contexts.

##### Measurements and Study Apparatus

A week before the interview, each physician received a toolkit to reflect on their clinical practice. The toolkit included a workbook with 7 daily assignments on their experiences and patient interactions as well as stickers featuring emotional words and contextual images to help express their thoughts and feelings.

##### Study Process

We asked physicians to reflect on their process for diagnosing patients and explaining treatment plans, especially in difficult situations such as delivering bad news. They were also asked to share strategies for handling uncooperative patients or those who rejected treatment. Physicians were encouraged to use the 2 sticker sets to respond to workbook questions or use them as interview resources. During the interview, physicians were guided through the workbook to discuss their specific approaches, experiences, motivations, stressors, and methods for negotiating treatment plans.

##### Analysis

In total, 2 researchers from the human-computer interaction (HCI) field and a cardiologist analyzed the interview data, with all recordings fully transcribed. The HCI researchers developed design strategies through thematic analysis, whereas the cardiologist reviewed and selected strategies for further evaluation in stage 2.

##### Results

Our initial interviews with 8 physicians produced 2 key outputs: 4 physician personas (*teamworker*, *minimum effort professional*, *the veteran*, and *self-actualizer*) and 7 design strategies for explainable prediction. Each persona included pseudodemographics and progress bars depicting their personalities across 4 parameters: *passionate/realistic*, *rookie/veteran*, *work-oriented/life-oriented*, and *individualism/collectivism*.

**Figure 1:**
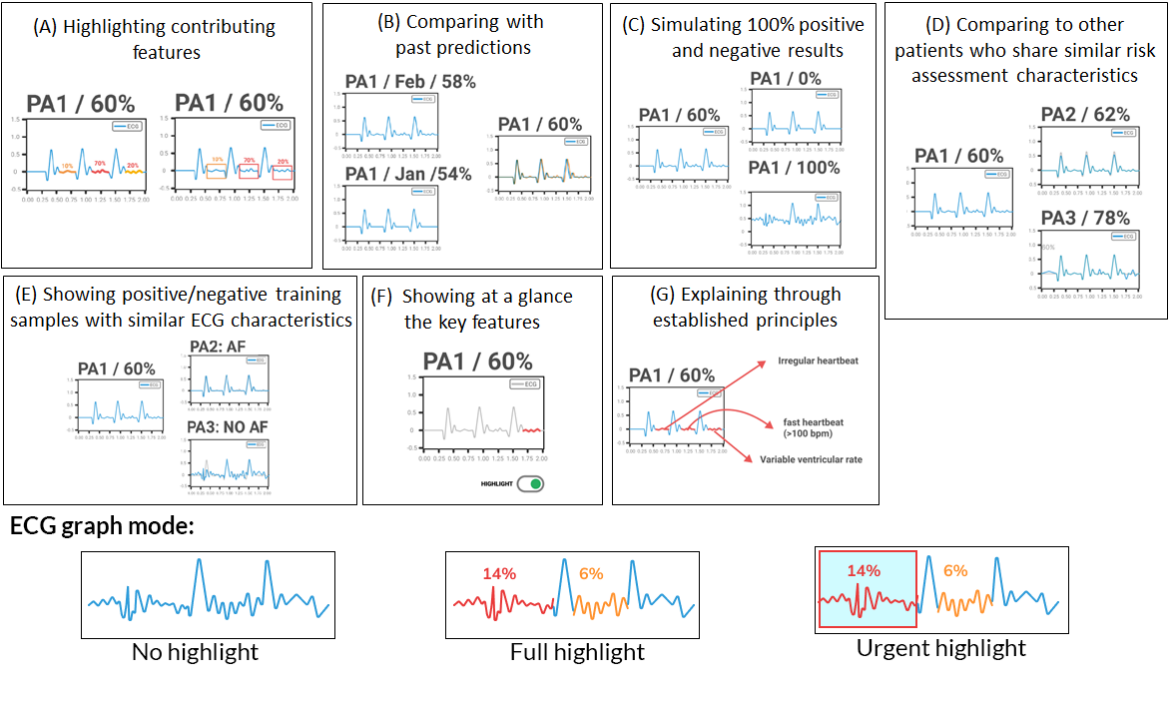
The 7 explainable artificial intelligence strategies (A-G) that were examined in the initial 2 stages of the study, expanded from the previous publication [39]. AF: atrial fibrillation; bpm: beats per minute; ECG: electrocardiography; PA1: patient 1; PA2: patient 2; PA3: patient 3.

The research team discovered that participants had common concerns about self-efficacy in patient treatment, feeling validated when treatments succeeded but disempowered when seen as incompetent. This insight led to the development of 7 design strategies for explainable prediction. Participants emphasized the need to justify decisions by clearly explaining issues such as ECG results to patients. We reviewed and adapted explainable AI strategies from previous studies [40] for cardiology. The seven strategies were (1) highlighting key ECG features (strategy A), (2) comparing with historical predictions (strategy B), (3) simulating extreme ECG outcomes (strategy C), (4) comparing with similar patients (strategy D), (5) showing similar training examples (strategy E), (6) providing an at-a-glance mode (strategy F), and (7) explaining through established ECG principles (strategy G; Figure 1 [39]).

#### Stage 2

##### Overview

In the second stage, we evaluated the feasibility of the 7 design strategies from the first stage, focusing on those that could be implemented using explainable AI. Physicians first described their usual process for assessing recurrence risk and discussed the potential role of predictive models and explainable AI. We then introduced the explainable AI strategies and gathered detailed feedback on which would most effectively clarify predicted outcomes for other physicians and patients.

##### Study Process and Findings to Guide the Prototype Development

We conducted 2 group design sessions with 8 cardiologists to critically review and assess our design strategies. Initially, 5 cardiologists were asked to evaluate the advantages and disadvantages of each strategy, describe scenarios in which they would be useful, and reflect on their concerns regarding implementing such explainable features.

From the 7 proposed strategies, we selected 4 (strategies A, B, D, and G) for further development into a web-based prototype using a high-fidelity explainable CNN model with real patient data. In total, 3 cardiologists reviewed the prototype using the concurrent think-aloud approach [41] and provided feedback. They also discussed concerns about using AI during posttreatment care, specifically on whether the explainable features enhanced their trust in the AI system and how the system might add or reduce value in their practice.

##### Participants

A total of 5 cardiologists with experience treating AF were recruited from Kyoto Prefectural University of Medicine to assess the explainable AI strategies. In addition, 3 cardiologists from the same institution were brought on to evaluate a web-based prototype.

##### Results and Study Apparatus Development

The 4 effective design strategies identified were highlighting contributing features (strategy A), comparing with past predictors (strategy B), comparing with other patients (strategy D), and explaining through established principles (strategy G). Participants’ choices were guided by 3 principles: discernibility (highlighting relevant noteworthy parts), comparability (intra- and interpersonal comparisons), and evidence-based approaches (derived from diagnostic examples or clinical literature). Strategies to avoid included non–evidence-based methods and those showing only partial graphs.

In general, physicians were highly receptive to the AF’fective prototype, particularly its explainable features, which they found useful for evaluating treatment effectiveness in posttreatment monitoring. On the basis of their feedback, we advanced to web-based prototyping.

**Figure 2:**
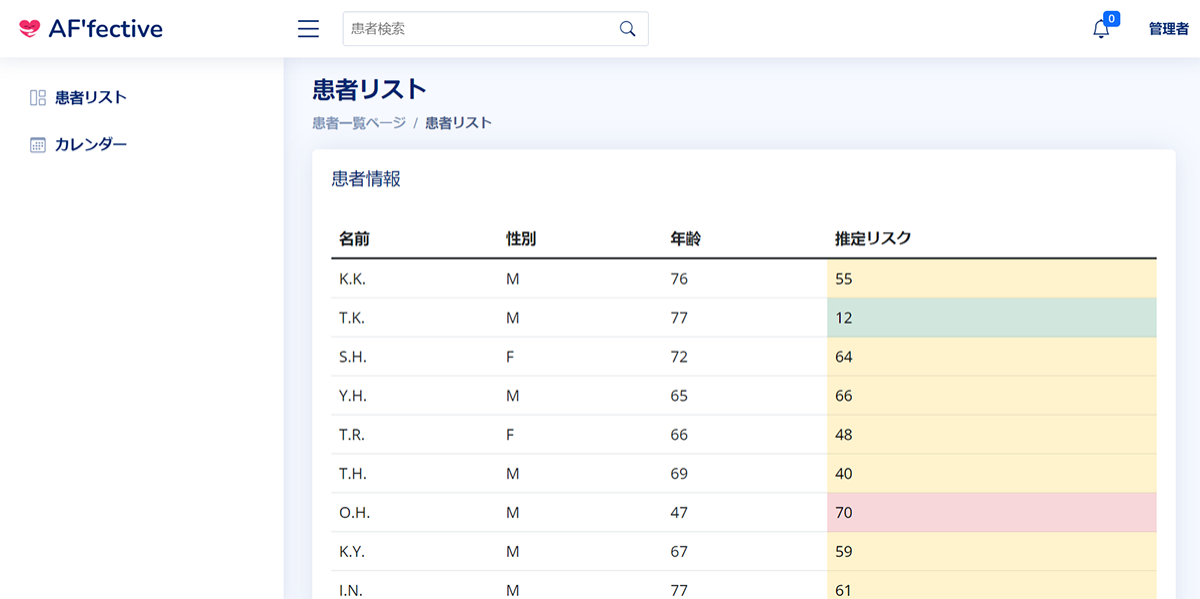
The patient overview screen.

**Figure 3:**
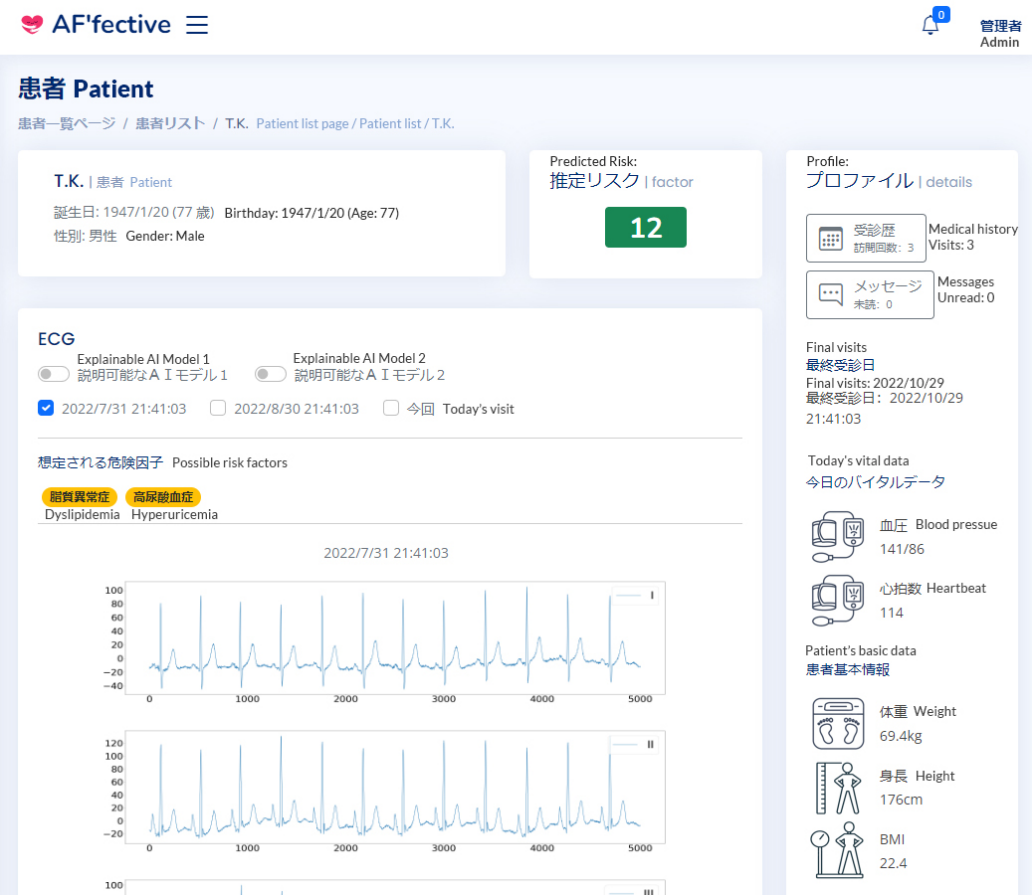
Screenshot of the patient page, including extra risk factors. ECG: electrocardiography.

The web-based prototype was developed using Figma (Figma, Inc), a popular tool among user experience (UX) designers for creating high-fidelity and web-based prototypes at minimal cost during the development process. Our prototype comprised 2 main screens: a patient overview screen and an individual health record dashboard (to avoid repetition of similar images, we share the screenshots from the final system in this paper instead; Figures 2 and 3).

The overview screen displays patients’ demographic details and predicted recurrence risks, allowing cardiologists to sort by various demographic and risk factors. The health record dashboard displays (comparable and evidence-based) health information gathered from a patient’s previous visits. Our predictive model will automatically extract and highlight keywords or risk factors based on the patient’s visits and health data. In addition, the model differentiates the importance of the risk factors and offers 3 tiers of ECG result presentation based on the level of urgency for physicians: no highlight, full highlight, and critical highlight only.

In critical highlight mode, only urgent health information is shown, whereas the full highlight mode displays all details, including less urgent AI highlights. Validated risk factors are listed as diagnostic keywords in the right panel, helping physicians quickly identify the main contributors to the predictive outcome.

## Methods

### Overview

Our research followed a 3-phase approach collaborating with stakeholders familiar with AF management. In the first phase, we conducted semistructured interviews with 8 cardiologists to explore potential explainable AI strategies for postablation care. From these insights, we developed 7 design concepts, which were then critically assessed and refined by another 8 cardiologists in the second phase. This process led to the selection of 4 explainable strategies. In this section (the final phase of the study), we built a functional prototype incorporating real patient data and invited a third group of 7 cardiologists to evaluate its effectiveness and feasibility in clinical settings.

### Ethical Considerations

This study received ethics approval from the relevant institutional review boards (IRBs) in Japan, including the Kyoto Institute of Technology IRB (2021-08) and the Kyoto Prefectural University of Medicine IRB (ERB-C-2014). Informed consent was obtained from all participating physicians, who volunteered to take part in the study without receiving any compensation. To ensure the privacy and confidentiality of patient health data, all personally identifiable information, including patients’ names, was removed and replaced with unique codes. This anonymization process ensured that no data could be traced back to individual patients.

### Stage 3

The web-based prototype from the previous stage was developed into a fully functional progressive web application (PWA) prototype, which was then tested and evaluated by cardiologists.

#### Patient Data

A total of 11 postablation patients consented to provide data for the prototype. Protected health information, such as names and birthdays, was masked or replaced due to IRB requests, but other health data (eg, ECG, body measurements, blood pressure, and visit history) were used to closely resemble the original patient health data for a realistic prototype.

#### Participants

In total, 7 cardiologists involved in the treatment of AF were recruited from the Kyoto Prefectural University of Medicine in Japan. All of them had experience diagnosing patients with AF and performing catheter ablation.

#### Measurements and Study Apparatus

In total, 3 widely adapted UX-related questionnaires were implemented in our study: the User Experience Questionnaire (UEQ), technology acceptance model (TAM), and Mobile App Rating Scale (MARS). The rationales for including these questionnaires are explained in the following sections.

##### UEQ Inclusion Rationale

The UEQ, developed by Laugwitz et al [42] in 2008, consists of 26 items across 6 dimensions: attractiveness, perspicuity, efficiency, dependability, stimulation, and novelty. The scale ranges from −3 (*most negative*) to +3 (*most positive*). Scores closer to 0 are considered “neutral.” A data analysis tool is provided for interpreting the results, with benchmarks in 5 tiers: excellent, good, above average, below average, and bad [43]. Our goal during prototyping was to achieve above-average ratings. The English version was translated into Japanese.

##### TAM Inclusion Rationale

Previous research suggests that a user’s intention to use is the primary predictor of actual system use [44]. To ensure that our PWA was both useful and usable in a medical context, we implemented the TAM to assess user acceptance and intention to use our explainable AI system. Originally developed by Davis et al [45], the TAM is a key model in new technology development. We adapted it for our PWA evaluation focusing on 3 dimensions: efficiency (perceived usefulness), ease of use (perceived ease of use), and intention to use (behavioral intention to use). For the TAM, a 7-point Likert scale is used, ranging from 1 (*strongly disagree*) to 7 (*strongly agree*). A score of 4 represents neutrality.

##### MARS Inclusion Rationale

Despite its name, the MARS is widely used to assess the quality of health applications across various types of digital platforms. It evaluates 4 dimensions: engagement, functionality, esthetics, and information quality [46,47]. We selected the MARS to assess the information quality of our PWA using the Japanese translation by Yamamoto et al [48]. The MARS uses a 5-point Likert scale ranging from 1 (*inadequate*) to 5 (*excellent*). Scores of >3 are considered above average.

#### AF’fective: The Web-Based PWA

**Figure 4:**
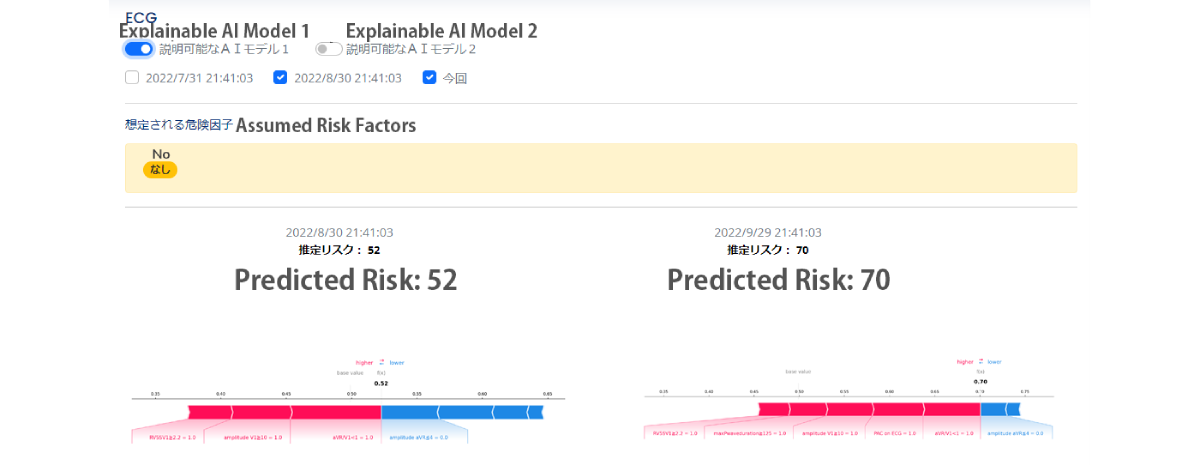
Screenshot of the explainable items and how they were displayed in the system—explainable model 1. ECG: electrocardiography.

**Figure 5:**
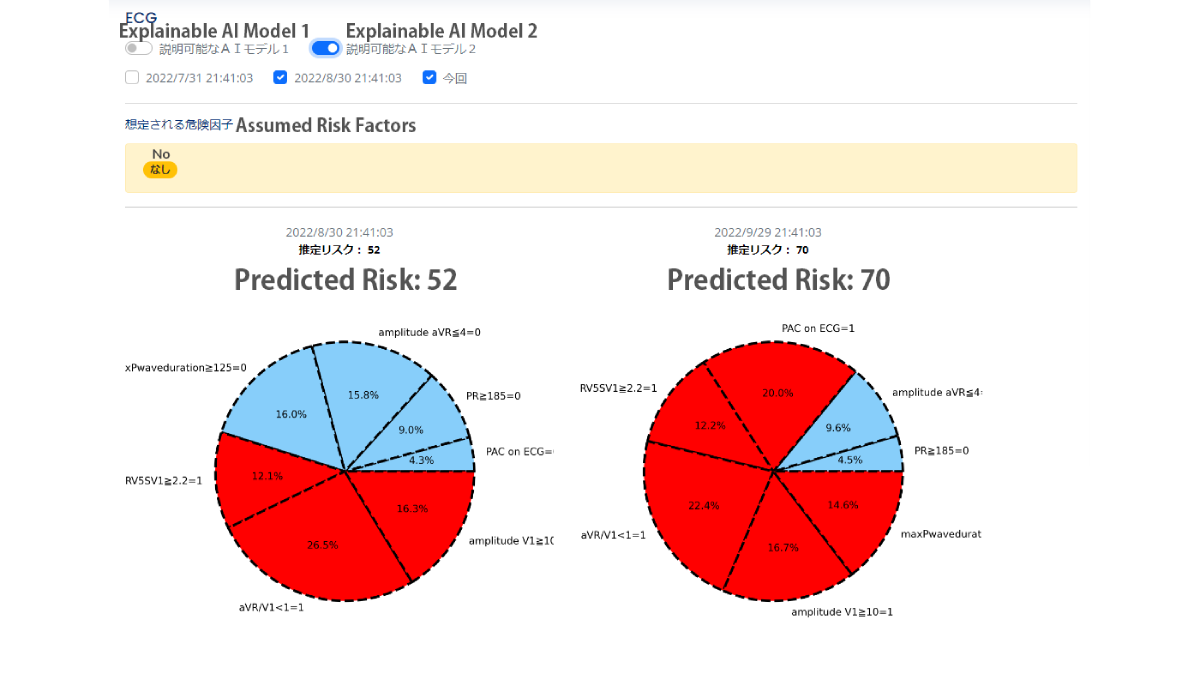
Screenshot of the explainable items and how they were displayed in the system—explainable model 2. aVR: Augmented Vector Right; ECG: electrocardiography. PAC: Premature Atrial Contraction; PR: PR Interval (the onset of the P wave to the start of the QRS complex).

A fully web-based PWA was developed using the Ionic framework (Drifty) for the front end and Python (Python Software Foundation) for the back end. Access was strictly controlled through an authentication system to ensure data security. The PWA included 2 main pages that were developed based on our explainable strategies: a patient overview screen (corresponding to strategy D) and an individual health record dashboard (corresponding to strategies A, B, and G). One noteworthy change was made to our predictive model, which also influenced our explainable features. On the basis of physicians’ feedback, we omitted direct highlights on the raw ECG graph in stage 2 (using Grad-CAM; see the demonstration in Figure 1 [39]) and, instead, used the 7-item features derived from the standardized clinical interpretation of ECG graphs (corresponding to strategies A and G in particular; see the demonstration in Figures 4 and 5). In particular, we replaced the explainable AI model used in previous stages with a SHAP model [49], which was constructed to highlight the extent to which different key clinical features—commonly used as established interpretation principles in AF prediction for ECG data (eg, maximum P wave duration and augmented vector right [aVR]/first precordial lead [V1])—contributed to the decision-making process for strategy G. Therefore, the predictive model was updated from a CNN model (image based) used in the previous 2 stages to a Cox regression model (feature based) [26] to accommodate the introduction of 7 standard features for prediction (and, later on, SHAP for explaining). While physicians were not shown the Cox regression model’s performance to avoid biasing them, it achieved strong evaluations, with an area under the curve of 0.72, sensitivity of 83%, and specificity of 58%. The model was trained on data from 502 patients, with an average follow-up of 6.2 (SD 3.5) years. A total of 13.1% (66/502) of the patients developed new-onset AF [26].

These standardized clinical explainable features are included in the following list. These features were derived from the interpretation standards that cardiologists commonly apply when reading an ECG graph and assessing a patient’s risk level. As indicated in our previous studies, while directly highlighting sections of the ECG graph that contributed to the predictive model’s output can explain the model’s prediction, it does not aid physicians in interpreting the graph. To enhance the interpretability and physician-friendly explanation, we asked cardiologists to standardize their interpretation of the ECG graph into higher-level features that are more straightforward and easier to understand for their fellow cardiologists. Our previous work [26] provides details on training a predictive model based on these new features:

1. Max P wave duration >125 ms: whether the maximum P wave duration is >125 ms
2. aVR/V1<1: whether the amplitude ratio of the P wave is <1
3. Amplitude V1≥10: whether the P wave amplitude in V1 is >0.1 mV
4. Amplitude aVR<4=0: whether the P wave amplitude in the lead aVR is <0.04 mV
5. PAC on ECG=1.0: whether there was one or more supraventricular ectopies during recording (removed in the latest model for simplicity from the work by Nishimura et al [26])
6. RV5SV1≥2.2=0: whether the amplitude (height) of the R wave in the fifth precordial lead plus the amplitude (depth) of the S wave in V1 is >2.2 mV
7. PR≥185=0: whether the interval between the P and R waves is >185 ms

The individual health record dashboard (as shown in Figure 3) also allowed physicians to check past visit ECG graphs and explainable features (corresponding to strategy B). The physicians can obtain an overview of the basic patient data in the top left corner and then proceed to the raw ECG graph. In the main graph section, they can choose between 2 types of explainable models (see Figures 4 and 5 for the corresponding graphs) and add data from the patient’s history for comparison. The right panel provides additional patient correspondence data and physical measurements such as weight and blood pressure. The standardized features made it easier to compare patients when physicians navigated the patient overview board (corresponding to strategy G).

We also developed 2 interfaces to summarize the explainable features. The first interface (Figure 4) was developed using SHAP, a popular tool for explaining the outcomes of machine learning models [49]. It features a single bar chart that shows the combined influence of risk and protective factors. The second interface uses a pie chart to show the relative importance of the 7 items and their risk or protective contributions to the predictive outcome (Figure 5). Cardiologists were advised to access it using a computer, although the site is also available on mobile devices, to simulate their normal use context.

#### Study Process

Each cardiologist participated in a 1-on-2 interview with an HCI researcher and a cardiologist. Before the interview began, the HCI researcher set the scene for the cardiologist using the following statement: “You have 11 patients that you are currently caring for. You can see a list of them in the AF’fective system, which indicates their risks levels for recurrence.” Participants were first briefed on the system and then asked to test it by imagining the following four scenarios: (1) viewing a patient’s status, (2) checking a patient’s ECG graph, (3) viewing explainable model 1, and (4) viewing explainable model 2. We then asked the cardiologists to complete the study measurements (UEQ, TAM, and MARS, as described in a previous section) and began the semistructured interviews. Each interview lasted 45 minutes to an hour. All the interviews were conducted in Japanese and fully recorded, including screen recordings of the participants testing the AF’fective PWA.

#### Analysis

For the survey data, we calculated the mean and SD for each item in all surveys except for the UEQ, which has its own analysis tool in Microsoft Excel (Microsoft Corp) format. We compared our mean scores for the TAM and MARS against the theoretical average scores to determine whether physicians experienced any usability issues related to the interface design, functionality, information quality, or accessibility.

For the interview data, we reviewed and analyzed the transcripts following the thematic analysis framework proposed by Clarke et al [50]. The analysis was carried out by 3 researchers, 2 from the HCI field and 1 with a cardiology background. As the 2 HCI researchers were more familiar with the analysis method selected and had a long history of conducting thematic analysis, the initial formation of the themes was first conducted by them. The HCI researchers first read through all the transcripts to familiarize themselves with the content and highlighted interesting quotes. The quotes were then summarized and independently interpreted as “topics of interest” by the HCI researchers. The researchers then grouped the topics into several potential “themes” and involved the cardiologist to approve and edit the themes. The cardiologist researcher then joined the evaluation to come up with the final findings that were deemed meaningful in both the HCI and medical fields.

## Results

### Overview

In the final stage, we recruited 7 male cardiologists (aged 31 to 55 years) with 8 to 30 years of cardiology experience. After the interviews, they completed the surveys, and we calculated the mean scores to identify potential usability or design issues. The results suggested no significant usability problems, and the prototype showed strong potential to motivate physicians’ intention to use it due to its ease of use, reliable information, and explainable functionality. The detailed survey results are reported in the following sections.

### UEQ Results: Positive Perspicuity

The results from the UEQ showed that perspicuity received a positive evaluation (mean 1.21, SD 0.96), whereas attractiveness (mean 0.56, SD 0.71), efficiency (mean 0.625, SD 0.27), dependability (mean 0.33, SD 0.27), stimulation (mean 0.5, SD 0.3), and novelty (mean 0.58, SD 0.27) received neutral evaluations. Perspicuity stood out as the most positively perceived aspect of the UX, suggesting that ease of use and clarity are strong points of the AF’fective system. The neutral evaluations for the other aspects (attractiveness, efficiency, dependability, stimulation, and novelty) indicated that physicians had average or mixed perceptions of these factors.

### TAM Results: Above-Average Technical Acceptance

In addition, the TAM measures showed that perceived usefulness (mean 4.22, SD 0.31), perceived ease of use (mean 5.13, SD 0.77), and behavioral intention to use (mean 4.77, SD 0.68) scores were all above the average (mean 3.5). The behavioral intention to use score indicated a strong intention to use the AF’fective system. These results suggest that the identified factors motivated users to engage with the system.

### MARS Results: Perceived High Functionality and Information Reliability

Finally, the results of the MARS showed above-average scores for functionality (mean 4.25, SD 0.42), esthetics (mean 3.61, SD 0.68), information reliability (mean 3.38, SD 0.54), and perceived impact on practice (mean 3.33, SD 0.76). These results indicated that users perceived high functionality and reliable information from the AF’fective system, whereas they rated its esthetics and perceived impact on practice as somewhat mediocre. We were particularly interested in the physicians’ high ratings for information reliability as these suggested that they felt confident in understanding and agreeing with the predictive model’s decisions. Notably, this rating was achieved without physicians knowing the predictive model’s performance. This serves as a strong indicator that insights derived from explainable models play a crucial role in physicians’ adaptation to our technology. This was deemed acceptable for a system in its prototype phase.

## Discussion

### Principal Findings

#### Overview

Aligned with our positive survey results, cardiologists expressed strong acceptance of the explainable features and willingness to use the system during the in-depth interviews. They also praised the system’s enhanced efficiency, comprehensibility, comparability, consistency, and trustworthiness:

I think it is useful to be able to express in a general, universal, numerical way what we used to say in the past, like, “This person is likely to have a recurrence,” by using a risk score. I think it is useful.

P04; male

In clinical practice, we honestly don’t have that much time to look at ECGs and scores in detail, so I think AI is a way to make it easier for us to get the numbers out.

P07; male

I think it is easy to pick up if you can tell from a quick glance at the list that a person in red is at higher risk of recurrence, or that a person in green has a low probability of recurrence.

P06; male

While most cardiologists in our study viewed the real data–backed explainable AI system positively, we identified potential concerns and (possibly) false expectations for its real-life implementation. During the interviews, cardiologists were asked to envision using the system in their daily routines, leading to a realistic discussion after 2 rounds of iterative design. This probing step was crucial for understanding the feasibility and challenges of implementing the system in clinical settings. Our findings are discussed in the following sections through 4 key themes.

#### Explaining the Symptoms Does Not Equal to Identifying the Causes

Perhaps due to our intentional mimicry of cardiologists’ methods for identifying irregularities in ECG graphs, participants showed high acceptance of the predicted results after viewing our 7-item AF clinical explainable features. In fact, P04 gave a very positive endorsement of why the explainable items were necessary:

I am of course curious as to why the risk was higher, so the AI [predictive] model would be rather meaningless without a graph [with explainable AI items] that can explain it, or perhaps it would make me wonder why the model [makes such a decision].

P04; male

According to P04, the predictive model’s results may fail to convince them without an explanation of why the model makes a particular decision. P04’s reaction suggests that physicians find explanations based on the new clinical explainable features both agreeable and trustworthy. However, a concern emerged regarding the limitations of these features. A key request was, “Then what should I do?”—cardiologists wanted to understand the features influencing the risk score and actionable strategies to address them. This aligns with previous insights showing a preference for clear indicators and actionable steps. In one case, P05 even suggested using the system to nudge patients toward behavior change:

We are at a stage where only these numbers [of explainable items] are available. 40 or something like this is the only thing that comes out, so what should I do [with each of the items]?

P04; male

I feel that the numbers alone don’t mean much, or don’t seem to lead to a change in [patient’s] behavior.

P05; male

It should be noted that physicians treated each explainable feature as a “treatable item,” which was not possible with an ECG graph–based explainable model. This improvement suggests that converting graphical highlights into standardized feature explanations can foster the expectation that addressing each item will reduce the risk score. In our current phase, it is still too early to determine how to further link our features with treatment approaches; however, this does indicate an interesting and operationalizable future implementation for explainable models.

Hence, in our current system, while these features influence the predictive outcome, clinical causal insights should be interpreted with caution. Our 7-item explainable features are based on standard ECG wave interpretation, identifying AF symptoms but not directly indicating underlying causes such as excessive drinking or sleep apnea. As noted in the document by Shapley [51], estimating causal effects from explainable items can be misleading. Cardiologists should be cautioned about this limitation and guided to set appropriate expectations for decision-making.

A key lesson from our interviews was the need for better curation of explainable features in the model, especially in clinical contexts. While various approaches provide transparency in AI decision-making [31,39,52,53], it is crucial that the chosen features are relevant, operationalizable, and actionable for medical professionals. This focus ensures that AI predictions are both understandable and practically applicable, enhancing postablation patient care.

#### Window for Interpretation: When 0% and 100% Recurrence Risk Does Not Exist

While a predictive model can theoretically provide risk scores from 0% to 100%, in real life, extreme scores such as 0% or 100% are unattainable. Participants noted that the possibility of such extreme scores diminishes the perceived realism and reliability of the model’s predictions:

Theoretically speaking, it has to be 0, so even if it’s low, it’s still bad. You can’t say 30 is a good thing. There is still a chance for a recurrence...So there are cases where it comes out as 0? (Cardiologist interviewer: That would be considered extreme score. Well, some might be zero, but I think the smallest is probably 3 or 4 [in the natural clinical contexts].)

P03; male

I’m not quite sure what [the risk score] means. There are a lot of questions about what 12 means and what we should think about it. It’s not 0 [even for a normal person], So are you saying that AF can happen to anyone? (Interviewer: I don’t think we can lower that number any further.)

P05; male

In a similar manner, participants also questioned whether a predictive score of 100% was at all meaningful in clinical practice:

*Are you saying that even if all of [the items] were full, it would not be 100?(Interviewer: Even if [all of the items were] full, I think it will probably be around 80-92, which is the highest risk score [in the natural clinical contexts].)* [P01; male]

It should be noted that it is uncommon for a predictive model to yield a full 100% score in clinical contexts, particularly in postsurgery contexts. The contributing factors would generally contain complex interactions and dependencies.

Cardiologists emphasized that recurrence risk scores should be viewed as reference points, not absolute indicators. Establishing a threshold, such as 50, is essential for guiding actions, but borderline scores (eg, 49 or 51) still warrant patient warnings or follow-ups. Similar “borderline windows” were noted in a grief-related study [52] where psychologists suggested taking the score as a reference for concerning cases. Although the width of the borderline window can also be highly subjective for each individual cardiologist and possibly for patients, this approach is common in clinical practice. Future studies should develop nuanced protocols for borderline cases to prevent misdiagnoses and patient stress, potentially standardizing risk level interpretation, as suggested by P05:

I think it would be better to indicate the risk of developing the disease on a 5-point scale, such as low, medium, and high, because I don’t think there is such a difference between 40 and 41.

P05; male

In treatment decision-making, another notable implication from our findings was the patient’s presumptive belief that the risk score should be 0%, particularly after undergoing ablation. It was apparently conflicting with what the predictive model would reveal:

However, if you think about it, after AF ablation, the number of people who think that AF recurrence is quite small. For example, more than 70% to 80% of the patients think it is zero.

P03; male

Even cardiologists familiar with the postablation risk might expect that addressing the explainable items (see the Methods section for more discussion on these items) could eliminate the recurrence risk:

Considering the importance of risk scoring...I am very interested in the explanatory factors or items involved, and which of them are [positively correlated to the risk score]. If the purpose of this system is to follow up the patient, it would be even better if we could get such risk factors and know what kind of intervention is being done here.

P01; male

It struck the research team that popular explainable AI approaches could lead to misinterpretations and unrealistic expectations, such as believing that a 0% recurrence risk is easily attainable. Our study highlights the need to set accurate expectations about what predictive results and explainable features truly imply in a clinical context and how much they can be influenced through intervention.

#### Offer a Holistic Risk Overview by Integrating Other Risk Factors and Temporal Data

In our study, we adopted the flow of cardiologists’ diagnostic process by reviewing ECG graphs and assessing the presence of known risk factors one by one. Therefore, other known risk factors, such as high blood pressure, smoking, and drinking, were included as additional “risk factors” alongside the predicted risk score derived from the patient’s ECG data. While we believed that this approach resembled a cardiologist’s normal decision-making procedure and would increase their acceptance of the model’s predicted results, our interviewees expressed concerns about the potential for the predictive model to yield biased risk estimates:

There are many known risks that are commonly associated with AF, such as high blood pressure, obesity, lack of exercise, etc... Inputting it would further make this risk [score], or perhaps, the evaluation, more correct.

P03; male

So we are predicting the risk score purely based on ECG...If you are going to make progress, I think it would be good to include obesity information, bmi, waist circumference, and so on.

P06; male

Furthermore, some of our participants also indicated that they would prefer a clear display and association with the medications or ongoing treatments that the patients are receiving. For instance, P02 requested us to explore whether the explainable model could reflect the effects of the patient’s medications, and P01 indicated that it would be better to see which interventions were done to lead to the risk score:

If there was information on arrhythmia medications, it would be more useful. I think the doctor might be able to think about it more holistically, such as, “This ECG variation [highlighted by the explainable model] is caused by the medicine the patient is taking.”

P02; male

If the purpose of this system is to follow up the patient, it would be even better if we could get such risk scores and know what kind of intervention is being done here.

P01; male

As the cardiologists pointed out, although our model provided an overview of the other risk factors, it would only be meaningful if they could establish a connection between the variation in risk scores and the specific risk factors. Our findings indicated that, for explainable elements to be truly useful in real-life practice, they need to relate to more holistic and treatment-related risk factors or medications. Otherwise, the contribution of such a predictive, even explainable, model to preventing disease recurrence would be quite limited.

In the end, perhaps the most important takeaway from the interviews was the need to provide a holistic overview and explanation of intrapatient temporal data and risk scores. Echoing the insights from previous interviews, the design strategy of comparing interpersonal ECGs (strategy G) was heavily criticized by the physicians as individual risk factors can vary significantly. However, comparing a patient’s current and historical risk factors could provide valuable insights into the evolution of these factors and potential trends in recurrence risk. For instance, participants P04 and P06 described how they would like to plan their interventions by tracking variations in risk scores and identifying the underlying causal factors over different time points:

For a patient with a high estimated risk this time, but lower estimated risk in the previous times, why did it become so high? What factors contributed to it? [I would expect] this AI model to estimate and explain.

P04; male

I think it would be better to look at it...from a longitudinal point of view...to see what kind of intervention I should use, what’s going wrong, and what the reason is for the increase [of the risk score].

P06; male

We were intrigued by the design opportunities revealed in our findings. As cardiologists reminded us, a significant and crucial aspect of their profession involves deciding on appropriate interventions. Although explainable AI models have shown promise in diagnosing and identifying patients at risk, they have yet to offer actionable insights for intervention planning. We believe that explainable AI tools should integrate comprehensive patient data, including temporal variations in risk factors, medications, and treatments, to enhance the model’s relevance and utility in real-world settings. In addition, the insights generated should be clearly linked to potential intervention strategies that could influence risk scores. Without such integration, the potential of explainable AI tools to contribute meaningfully to patient care, particularly in preventing disease recurrence, may remain limited.

#### A Sense of Control and Learning From the Model

The findings of our interviews revealed that cardiologists valued the sense of control and the learning opportunities provided by these models, aligned with the findings in the previous rounds that suggested a self-actualizing and constant learning side of cardiologists.

One of the key insights that emerged from our study is the potential of explainable AI models to help cardiologists prioritize patients at higher risk. As P04 addressed, there was a potential for these models to become an integral part of daily practice:

If it becomes a habit, I will probably look at people with high estimated risk and wonder why they were at higher risk.

P04; male

Moreover, P02 also offered a similar thought regarding triaging patients based on the risk scores:

So the follow-up should be more thorough, and those who are more likely to be affected [by the recurrence risk] should be followed up in a shorter period of time.

P02; male

By discriminating the patients that need urgent attention, physicians gain a sense of control over who to allocate more time and treatment resources to. Cardiologists indicated that such a potential new routine would enhance their work efficiency and allow them to spare cognitive capacity for more critical treatment planning. Interestingly, our interview findings suggest that cardiologists may appreciate useful “hints” or “interpretations” from explainable models to help them narrow down potential problems:

I understand that the ECG is used as the reference, but I think it would be easier to understand if there was an explanation of what is of interest in this ECG, even though those who understand may [already] understand it.

P01; male

The power dynamic between cardiologists and the AI model is worth exploring. To what extent should an AI model’s interpretations be considered, and to what extent should cardiologists accept its suggestions? These boundaries are critical to explore to ensure that the final AI models can be effectively integrated into the patient care routines.

Further enhancement of control over the prevention procedure might also occur as cardiologists begin to learn more from AI-augmented interpretation and actionability. Interestingly, the explainability of AI models appears to increase cardiologists’ desire to validate their treatment approaches:

So, at the time of the ablation, if I burn the tricuspid valve, you know [the score will go down], or if I burn the superior vena cava, you know [the score will go down]...you can learn more and more from time to time.

P03; male

I would take another ECG before prescribing too much medication, and see if [the risk score] goes up or down on the next ECG...For those in yellow...I will adjust my [treatment plan] according to the risk level of the next ECG.

P06; male

P06 went further and emphasized that, while AI models are valuable for providing quantitative assessments, their true worth lies in delivering actionable insights that can guide clinical decision-making. This sentiment reflects a broader interest among cardiologists in understanding how AI models arrive at their conclusions, particularly in relation to identifying intervention points:

I wonder if you are highlighting these risk factors to tell us these are the areas for intervention. I wonder if there is a reason for this risk factor, such as if we treat these factors, it could lower the risk.

P06; male

In general, our findings indicate that cardiologists, particularly self-actualizers, are not only open to integrating AI suggestions into their decision-making processes but also eager to enhance their knowledge through interaction with the AI model provided that these suggestions are accompanied by clear rationales and practical recommendations.

### Implications of Creating a Software as a Medical Device

In the development of AI algorithms for clinical applications, it is crucial to not only meet the user interface requirements but also ensure compliance with regulatory standards. Software as a medical device must adhere to international standards such as International Organization for Standardization (ISO) 13485 (medical device quality management systems), ISO 14971 (medical device risk management), and ISO 62304 (software life cycle processes). In addition, compliance with Food and Drug Administration approval and the European Medical Device Regulation is essential from the perspectives of patient safety, effectiveness, and legal requirements.

While the AF’fective study provides valuable foundational knowledge on user interface, it is still considered in the early stages of technology readiness level (1-3). However, with advancements in AI technology, the insights from this research could contribute to the revision of existing standards, such as the International Electrotechnical Commission 62366-1:2015 (usability engineering for medical devices). Specifically, the consideration of explainability in the AI result display methods proposed in this research—such as visually clarifying the rationale behind suggestions and intuitively conveying risk and uncertainty in interface design—will play a crucial role in helping health care professionals accurately understand AI-generated proposals and integrate them into clinical decision-making. Moreover, our study highlighted that health care professionals do not need to fully comprehend the technical background or mechanisms of AI. Instead, it is crucial to create a state in which users can reasonably understand “why this result was reached,” which is effective in building trust in AI. Such efforts extend beyond display design and have the potential to be incorporated into new standards that facilitate smooth communication between AI and health care professionals.

### Conclusions

This paper reports a 3-stage study involving 23 physicians to co-design, assess, and pilot an explainable AI system for AF postablation monitoring called AF’fective. Our co-design approach effectively identified 4 feasible explainable strategies. Furthermore, while we actively used explainable AI to identify the features contributing to the predictive results, our explainable strategies enabled the design of a more holistic patient monitoring system that incorporates contextual factors such as patient history and established principles to support physicians’ decision-making rather than merely explaining the features indicated by ECG graphs. In the third stage, the system prompted cardiologists to envision its use in their routines, leading to highly contextualized feedback. This study highlighted the need to curate actionable explainable features and set correct expectations for interpreting predictive scores. In addition, cardiologists were interested in understanding the AI’s reasoning and identifying strategies to address recurrence risk factors.

Interestingly, although our focus was on AF recurrence prevention, many insights appear generalizable to other disease care contexts, such as temporal risk score monitoring and setting realistic expectations for scoring, particularly as extreme scores (0% and 100%) are practically unattainable. Further investigation is needed to determine whether these insights can be universally applied to enhance the feasibility of implementing explainable AI in clinical settings.

A future study will build on these findings and further evaluate the potential challenges and advantages when implementing the AF’fective system in near-live or live clinical care routines. We believe that AI possesses the potential to revolutionize medical practices and we can only realize it through putting the technology in actual use case scenarios.

## Abbreviations

AF: atrial fibrillation
AI: artificial intelligence
aVR: augmented vector right
CNN: convolutional neural network
ECG: electrocardiography
Grad-CAM: Gradient-Weighted Class Activation Mapping
HCI: human-computer interaction
IRB: institutional review board
ISO: International Organization for Standardization
MARS: Mobile App Rating Scale
PWA: progressive web application
SHAP: Shapley Additive Explanations
TAM: technology acceptance model
UEQ: User Experience Questionnaire
UX: user experience
V1: first precordial lead
